# Supplementary figures and images for: The degree of polymerization and sulfation patterns in heparan sulfate are critical determinants of cytomegalovirus entry into host cells
Source: PLoS Pathog. 2021 Aug 5;17(8):e1009803. doi: 10.1371/journal.ppat.1009803 (PMC8384199; doi:10.1371/journal.ppat.1009803)

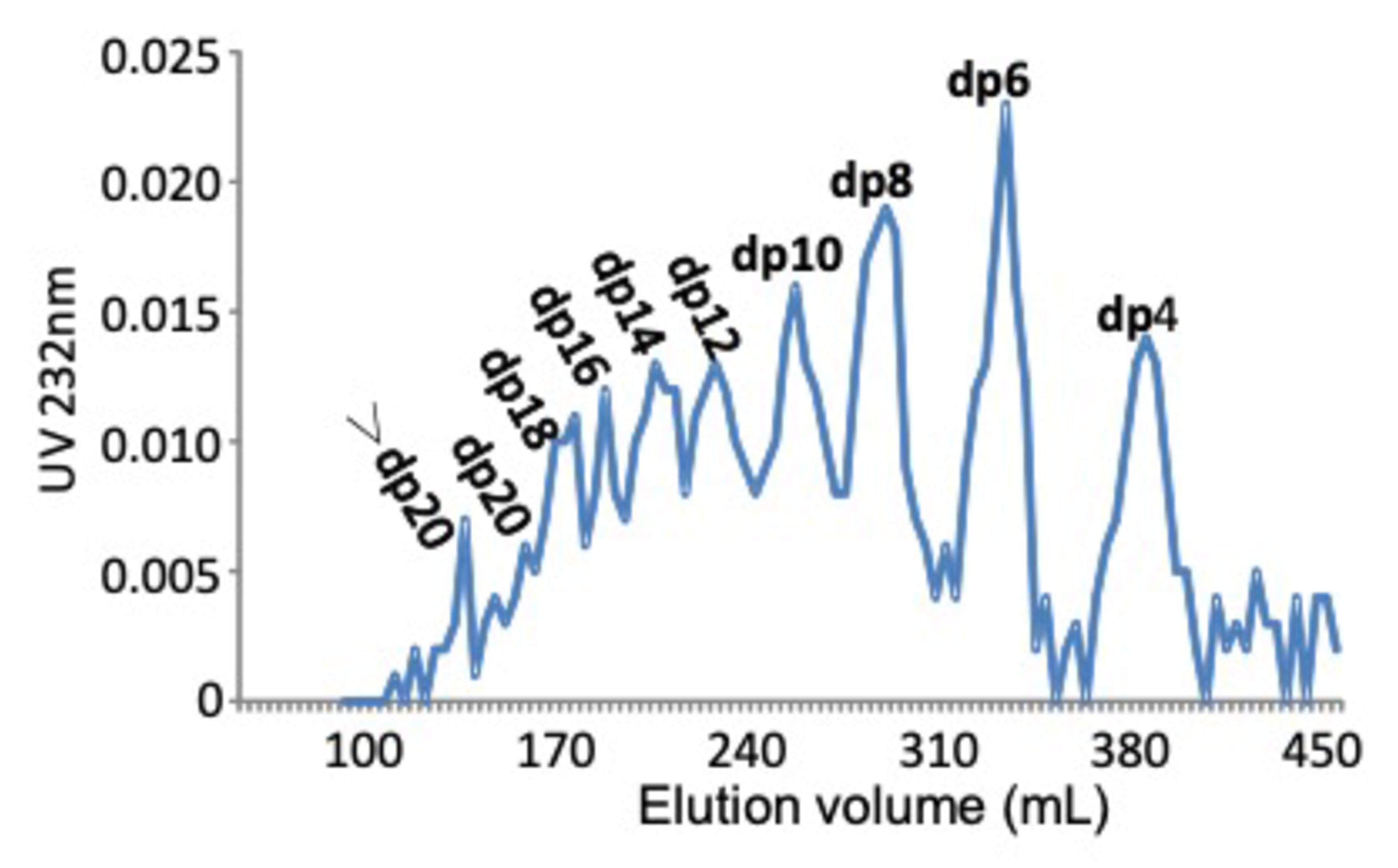

Supplement: S1 Fig — Fractions were collected and UV readings at 232 nm were taken for each fraction to reconstruct the chromatogram. Samples were pooled to obtain the oligosaccharide fractions of the desired size. (TIF) [file ppat.1009803.s001.tif]

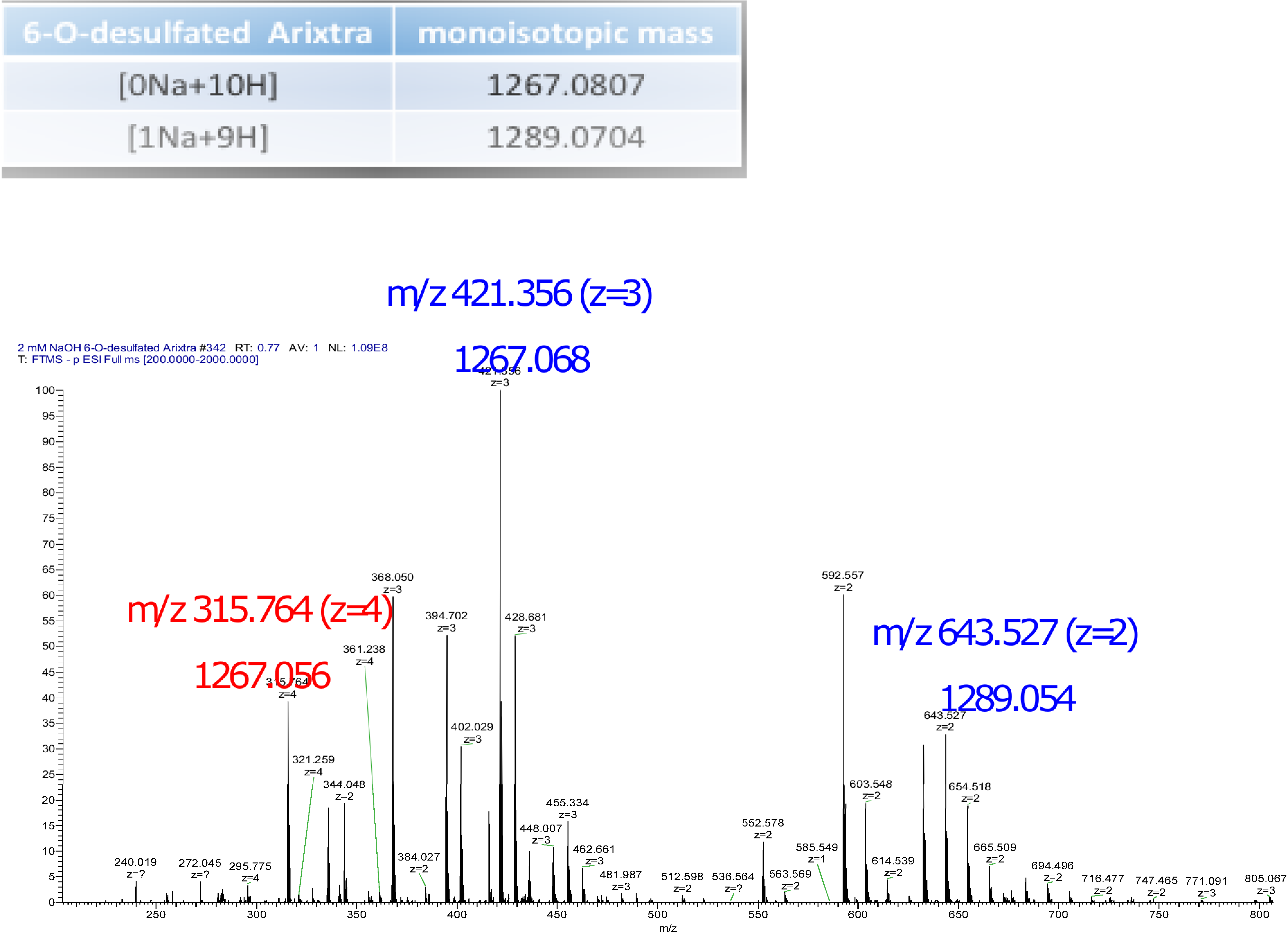

Supplement: S2 Fig — The most abundant MS masses were consistent with the loss of the three 6-O-sulfates from Arixtra. (TIF) [file ppat.1009803.s002.tif]

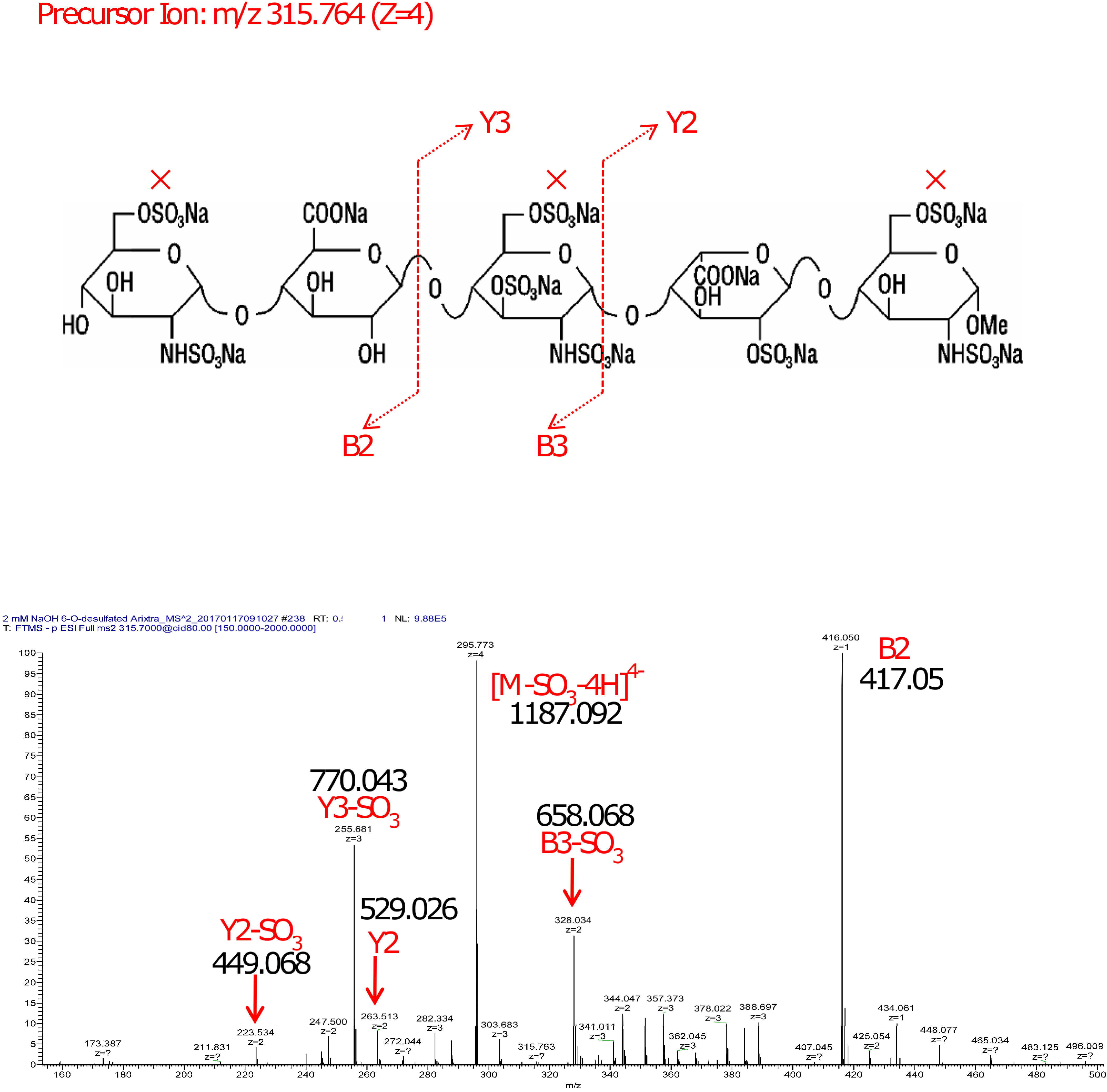

Supplement: S3 Fig — Glycosidic bond cleavages isolate desulfation to one desulfation event in the two non-reducing end residues; one desulfation event in the two reducing end residues, and one desulfation event in the central GlcNS. This pattern is consistent with 6-O-desulfation. (TIF) [file ppat.1009803.s003.tif]

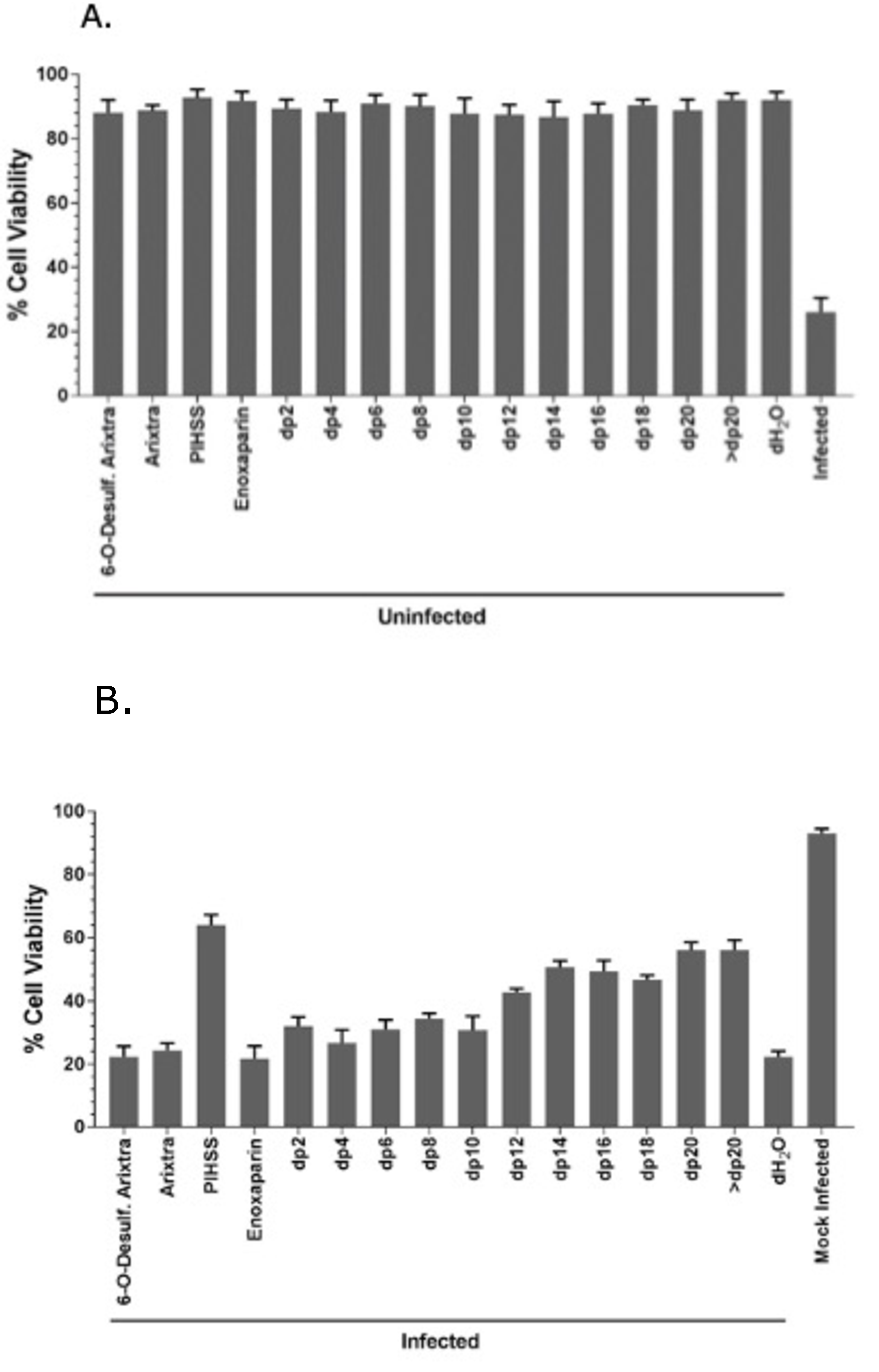

Supplement: S4 Fig — Primary HFF were pretreated for one hour with 10 μM of 1) 6-O-desulfated Arixtra, 2) Regular Arixtra, 3) Heparin sodium salt from porcine intestinal mucosa (PIHSS), 4) Enoxaparin, or series of heparin oligosaccharide from enoxaparin: 5) dp2, 6) dp4, 7) dp6, 8) dp8, 9) dp10, 10) dp12, 11) dp14, 12) dp16, 13) dp18, 14) dp20, 15) > dp20 or control (dH2O). Cells were either mock infected (A) or infected with HCMV (Towne strain) virus at an MOI of 3.0 (B) in the presence of test glycosaminoglycans. Cells were harvested at 5 days post-infection and cell viability was assessed using Trypan Blue exclusion assay. Results are representative of three independent replicates. Standard error of mean was plotted as error bars. (TIF) [file ppat.1009803.s004.tif]

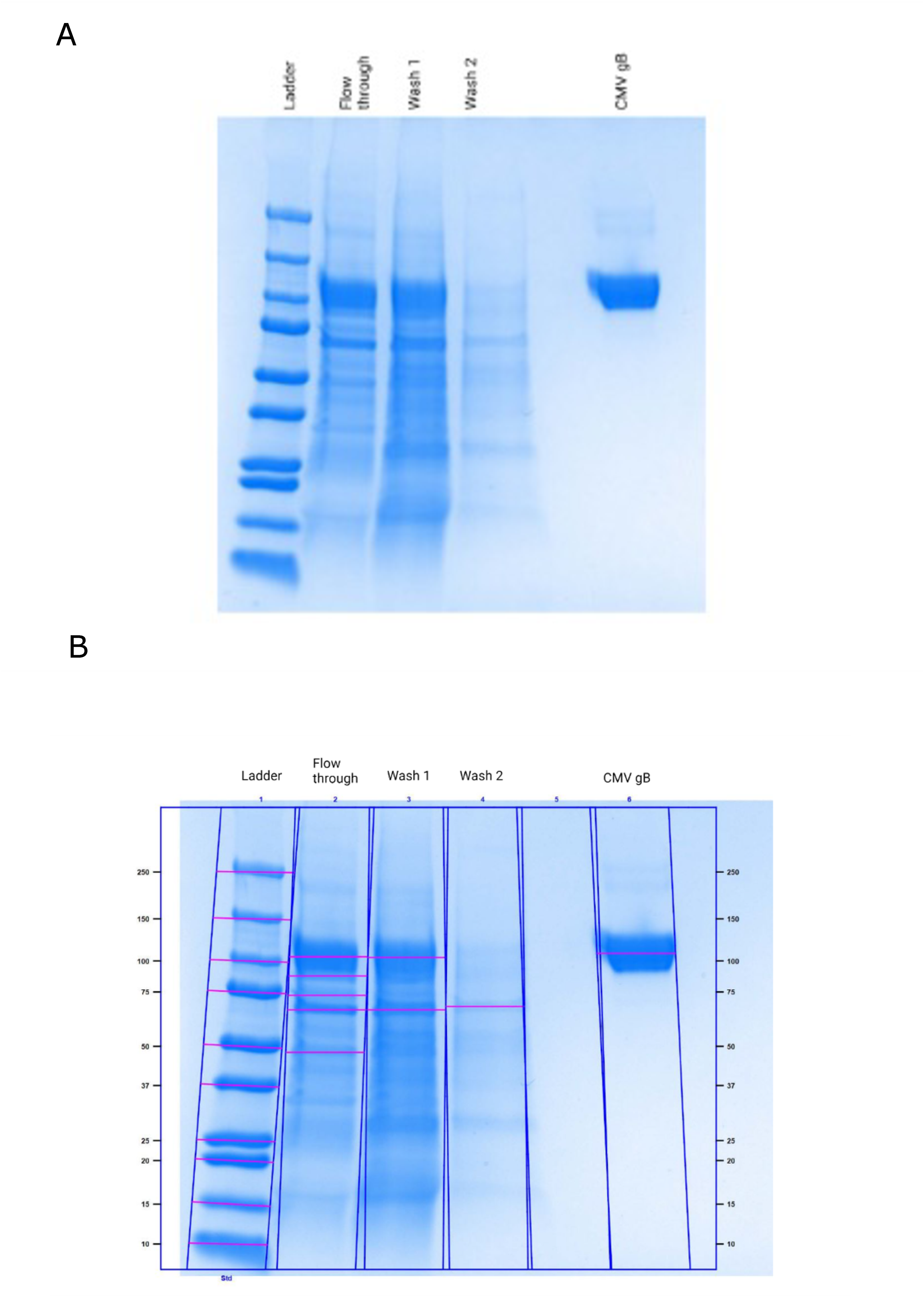

Supplement: S5 Fig — HCMV gB was expressed and purified for GAG binding assays as described in Materials and Methods. Protein purification was verified by SDS-PAGE followed by staining with Coomassie Brilliant Blue. (A) Coomassie protein gel of purified CMV gB as well as flow thorough and washes. (B) CMV gB protein gel lane analysis was performed by using Bio-Rad Image Lab Software. Coomassie purity of gB was determined to be 100% and molecular weight was 107.59 kD. Lane 1—protein ladder (Bio-Rad Precision Plus Protein Dual Color Standards), Lane 2—CMV gB column flow through, Lane 3—1st wash, Lane 4—2nd wash, Lane 5—blank, Lane 6—CMV gB. (TIF) [file ppat.1009803.s005.tif]
