# Supplementary material for: The degree of polymerization and sulfation patterns in heparan sulfate are critical determinants of cytomegalovirus entry into host cells
Source: PLoS Pathog. 2021 Aug 5;17(8):e1009803. doi: 10.1371/journal.ppat.1009803 (PMC8384199; doi:10.1371/journal.ppat.1009803)
Supplement: Attachment — Submitted filename: PLoS Pathogens Review 07092021.docx [file ppat.1009803.s006.docx]

Reviewer #1: This manuscript uses novel glyco arrays, peptides, and sulfation enzyme knockout lines in an attempt to map whether the degree of sugar polymerization and/or sulfation is important for MCMV or HCMV entry.

Reviewer #2: The authors have tried to demonstrate that structural specificities including sulfation patterns within heparin/Heparan sulfate play important roles in CMV entry and infectivity. While the area of research may be significant, many additional experiments are needed to improve novelty and reduce confusion and self-contradiction.

Reviewer #3: The manuscript by Hasan et al addresses the role of specific cellular glycan modifications to infection by human cytomegalovirus. Glycan modification of mammalian cell surface proteins are complex with a tremendous variation in the lengths of the polymer chains and the chemical linkages between the different carbohydrate moieties. Many viruses use glycan modifications as receptors and the various branched structures can have major impacts on tropism; specific types of structures being characteristic of specific tissues or even mammalian species. The general view is that herpesviruses use heparin sulfate glycan modifications for initial adsorption, which then facilitates the binding to specific entry receptors (e.g., nectin for HSV). Here, Hasan and colleagues characterize the use of HS for cytomegalovirus infection, focusing on the polymer length and the sulfate linkage. They use an array approach to measure the binding of CMV to a variety of synthetic glycosaminoglycans and hone in on specific heparan sulfate characteristics for further study. Further experiments showing that soluble HS can interfere with CMV infection, and experiments using cell lines deficient in the enzymes that generate specific sulfate linkages lead the authors to conclude that the CMV bind principally to HS comprised of longer polymer chains bearing a 3-O-S sulfate linkage. Authors’ then speculate on the significance of this conclusion suggesting potential use in development of antiviral therapeutics.

A number of concerns outlined below limit enthusiasm for the study. In summary: 1) Experimental rigor is low given the lack of appropriate and rigorous statistical analyses of significances (especially in cases where the measured differences are small.) Moreover, some experimental protocols do not seem well matched to the hypotheses and this creates valid, alternative interpretations (see below for more detail). Together these concerns cast doubt on the validity of the conclusions. 2) The studies were conducted with only the “Towne” strain of HCMV. Given what is now appreciated about the genetic diversity of HCMV in vivo, including additional strains would increase the impact of the study. As a related point, there was not effort to identify what HCMV glycoprotein(s) are involved in the proposed interactions. Prior research has implicated gB and gM/gN in binding to GAGs, but in neither case has this been extensively studied. Both glycoprotein complexes exhibit some degree of genetic diversity. 3) A better discussion of the benefits and drawbacks of developing therapeutics targeting this stage of HCMV infection would increase the impact of the study. - Brent Ryckman (University of Montana)

We appreciate the comments by the reviewers and believe they have helped us improve the quality of our initial submission. Answers to specific comments are listed below and major changes are summarized here:

1. We designed a CMV glycoprotein B construct, synthesized and expressed it in eukaryotic cells and studied binding with different glycosaminoglycans using Surface Plasmon Resonance (SPR). The results (Fig. 6) show that purified gB strongly binds to heparin; the competition assays with heparin analogs show that unmodified heparin and a 6-desulfated form of heparin compete with the gB-bound heparin significantly; however, Enoxaparin and its desulfated homologs are unable to compete. These results confirm the results in our GAG screening assay that degree of polymerization and sulfation of heparin had a strong effect on binding, and that 6-O sulfation is not required for binding but does increase affinity (Fig 1B, Table 2).
2. We performed statistical analysis on all relevant data where the differences were not apparent. Data was analyzed by ordinary one way ANOVA with multiple comparisons comparing the means of each test sample with control, and corrected using Dunnett’s post hoc test. Differences were considered significant if p<0.05.
3. We removed the figures and experiments that did not contribute significantly to the manuscript (schematic of heparan sulfate synthesis and peptide experiments).

**Part II – Major Issues: Key Experiments Required for Acceptance**
Please use this section to detail the key new experiments or modifications of existing experiments that should be absolutely required to validate study conclusions.

Generally, there should be no more than 3 such required experiments or major modifications for a "Major Revision" recommendation. If more than 3 experiments are necessary to validate the study conclusions, then you are encouraged to recommend "Reject".

Reviewer #1: 1) needs to have the full panel of mouse GAG modification knockout lines tested.

A total of 13 mutant cell lines were tested by the co-author (Lianchun Wang) for structural functional relationship of HS (PMID: 30377379). Several of these cell-lines are mutated for enzymes that are required for synthesis of basic HS structures. We hypothesized that specific sulfation of HS moieties is required for CMV entry. The specific sulfation pattern (the ligand binding site) is mainly determined by the added sulfation at the later stage of HS biosynthesis including both 6S and 3S, especially 3S which is rare and normally and generate highly specific, high affinity binding site. Thus we initially analyzed the HS3st serial mutants that perturb specific sulfation of HS and have been implicated earlier in HSV-1 entry (Fig 4). Later on we tested the entire library but only selected the serial Hs3st and Hs6st mutant cell lines that survived the virus infection. These cell lines are represented in Fig 5.

2) Characterization/quantification of the purity of their different fractionation experiments.

Fig. S1 shows size exclusion chromatography of enoxaparin separation and Fig. S2 shows ESI-MS characterization of 6-O desulfated Arixtra. Fig. S3 shows MS/MS analysis of the -4 charge state of Arixtra-3SO_3_. The pattern is consistent with 6-*O* desulfation.

3) Inhibition analysis with different tropic strains of HCMV.

Different tropic strains of HCMV are known to differ in the presence or absence of a pentameric glycoprotein complex. However, glycoprotein B is highly conserved across HCMV strains. Our new data shows strong binding of heparin to gB confirming earlier studies with HSV-1. We designed the gB construct based on a clinical (Merlin) strain of HCMV whereas most other experiments used a Towne strain, thus incorporating different tropic strains for experiments.

Reviewer #2: Fig. 1 is unnecessary. Similar or identical figures exist in many papers including at least one by the authors here (PMID: 22773448). Please just refer to the published paper(s).

We have removed Fig. 1 and inserted the citations.

Figs.2/3, this data will be more meaningful (informative) if binding to gB was also measured in parallel and compared with the data shown here. This would also allow the authors to measure the binding affinity more precisely, something that has been hard to achieve in the field.

As mentioned above, we designed a CMV glycoprotein B construct, synthesized and expressed it in eukaryotic cells and studied binding with different glycosaminoglycans using Surface Plasmon Resonance (SPR). The results (Fig. 6) show that purified gB strongly binds to heparin; the competition assays show that only heparin itself and a 6-desulfated form of heparin compete with the gB-bound heparin significantly. Enoxaparin and its desulfated homologs were unable to compete with heparin. These results confirm the results in our GAG screening assay where the sulfated forms of heparin were most effective in blocking virus infection.

Also, how do heparin structures (sulfation patterns etc.) compare with HS structures present in the glycoarray shown in Fig. 3?

Heparin and heparan sulfate (HS) are very similar molecules and the both consist of linear chains of repeating disaccharide units of glucosamine and uronic acid. During the HS/heparin maturation pathway, each step is dependent on the previous biosynthetic reaction. Modifications in various regions in these steps often lead to structural heterogeneity in HS and heparin molecules. Heparin consists mostly of L-iduronic acid whereas majority of the uronic acid in HS is d-glucuronic acid epimers. The average Heparin disachharide contains ~ 2.7 sulfate groups and HS contains ≥1 sulfate groups. HS chains are generally longer than heparin chains. The average molecular weight of HS and heparin is ~30 kDa and ~15 kDa respectively.

Fig. 3, instead of Fig.1 the common heparin and HS structures (disaccharide or higher) that contribute to binding should be shown.

We have removed figure 1 and inserted the citations that refer to HS and heparin structures in details.

Fig. 4, the results are confusing and correlation with heparin/HS structures is not well described. Higher polymerization means higher negative charge availability to bind positive residues on gB or viral envelope, nothing specific or truly novel here. If the point is to show that heparin blocks viral growth then that point was already shown by Teresa Compton and colleagues almost three decades ago (PMID: 83847). Figs. 5/6, argument similar to above (#4) applies here. Also, what is the new message here? Basically, multiple repetitions of results from Fig. 4. Not very impressive inhibition of infection. No statistics is performed to determine the significance.

The results of the original Fig. 4 and Fig. 5 (now Fig. 2 and 3) actually indicate the opposite of what the reviewer infers. The major point we wanted to make with the current Fig. 3 is that virus inhibition was not due to a simple increase in the number of potential HCMV binding sites per molecule of GAG. This can be observed by comparing results from Fig. 3 A and B (where the amount of available negative charge varies directly with degree of polymerization) with those from Fig. 3 C and D (where the amount of available negative charge is constant, as all doses are at equivalent g/L rather than mol/L). As evidenced by similar trend of inhibition obtained when treating cells with equivalent µM or g/L concentrations of GAGs, the simple amount of negative charge available does not explain the decrease in titer at increased degrees in polymerization. A preferred explanation is a minimum ligand binding size needed to inhibit HCMV growth, and/or avidity effects from multiple binding sites increasing the apparent affinity of GAG chains able to engage more than one site. Additionally, our data indicate that 6O-sulfation is less important to HCMV binding than N-sulfation, even though the charge density is similar between to two selectively desulfated products. These points have been expanded in the discussion. We wanted to emphasize the trend rather than differences between individual treatments, thus the data has been analyzed by ordinary one way ANOVA with multiple comparisons comparing the means of each test sample with control, and corrected using Dunnett’s post hoc test. Differences were considered significant if p<0.05. Fig 4 (now Fig. 2) showed preliminary screening using virus-GFP as a marker, which is indicative of virus entry, whereas Fig. 5 (now Fig 3) analyzed virus titers to show an overall impact on virus replication.

Figs. 8/9, the results do not compare well. While the virus replicates the worst in 3OST1 KO cells (Fig. 8) it shows the best entry in the same cells (Fig. 9). Contradictory results, what is going on? Also, why does the double knockout support better replication (Fig. 8 statistical analysis suggests that results are significant)? Structural analysis of the GAGs from the double KO should be made and compared with single KOs.

These are two different experiments, and the results aren’t necessarily contradictory. One experiment looks at the virus titers at 3 to 6 days post infection whereas another experiment is analyzing virus immediate early gene expression at 2 hours post infection as a surrogate for virus entry. The time difference between these two events is very wide. The comparisons have been made with the wild type cells in both cases and no claims have been made regarding the relative importance of one mutation over another. In fig 8 (now fig 4), all mutants were found to significantly impact virus titers compared to the wild-type and in fig 9 (now fig 5), all tested mutants were found to impact HCMV entry.

Fig. 10, what is the purpose or novelty of this experiment? Similar use of anti HS peptides has been demonstrated against CMV in multiple papers by Tim Sparer’s group.

We have removed this figure since it did not add significantly to the manuscript.

Reviewer #3: Suggested new experiments to strengthen the study.

• Some indication of that HCMV glycoprotein(s) are involved in the interactions proposed.

We have performed GAG binding studies with gB and included the new data.

• I don’t understand why the experiments in Figs 4-5 were done by pre-incubating the cells with the GAG derivatives. If the hypothesis is that the virus binds to these GAG structures on the cells, then would it not make more sense to pre-incubate the virus with the GAGs to “block” the sites on the virus before you add the virus to the cells (Like using soluble receptor). This would also lessen the likelihood of effects on the cell physiology.

These experiments were designed to mimic the real-world scenario where patients would be given a drug for potentially prophylactic applications. In this case, host cells would first see the drug and not the virus.

• Showing “dose responses” in experiments such as those in Figs 4-5 (potentially others) would also help strengthen the conclusion of preferential binding to specific GAGs.

We initially optimized the doses for optimal effect however dose-responses were not calculated. This is something we plan to do in future for the selected GAGs.

**Part III – Minor Issues: Editorial and Data Presentation Modifications**
Please use this section for editorial suggestions as well as relatively minor modifications of existing data that would enhance clarity.

Reviewer #1: Really arrays in Fig. 2/3 could be combined into 1 figure A and B. Figure 3 is a just a more detailed analysis of the HS array in figure 2.

We have combined these two arrays in new Fig. 1.

Previous publications do not indicate that CS or DS affect HCMV binding. Why does this array disagree with this? Length of the GAGs or sulfation? How much do these arrays relate to what is seen on actual susceptible cells?

Binding of CS was limited to the highest dp of only CS-D, a CS chain that is primarily sulfated at the C-2 position of GlcA and the C-4 position of GalNAc. CS-D has a much higher sulfate density compared to CS-A/CS-C (which are primarily only monosulfated at the GalNAc at the C-4 and C-6 positions, respectively), and the 2-O sulfation of the GlcA is similar to the common 2-O sulfation of IdoA found in heparin. Dermatan sulfate also only bound significantly in the highest dp tested. While DS has a sulfate density similar to CS-A/CS-C (one sulfo group per disaccharide, found at the GalNAc), it also bears similarity to heparin in that it has IdoA in its disaccharide repeat as most commonly found in heparin, unlike CS which has GlcA (differing by epimerization at the C-5 position). We would hypothesize that it is these heparin-like qualities that causes CS-D and DS to bind at high dp.

Fig. 5 the degree of reduction is only 1-1.5 log reduction in titers at the highest dp. Although statistically significant, it also means that ~90% of the virus is NOT dependent on the dp and or other entry mechanisms.

1-1.5 log reduction means that >90% of the virus is dependent on the HS. (e.g. 10 particles vs. 100 particles is a log reduction.)

Fig. 7 could be moved to supplemental data as this would not be expected to cause cell death. Important but not necessary in the body of the paper.

We have moved Fig. 7 to supplemental data (Fig S4).

Fig. 8 These MCMV cell lines only show a modest reduction implying multiple HS/modifications are important for infection. There are a whole panel of the these lines available from the Wang /Esko lab in order to provide a more complete picture of the important of the different sulfations on viral entry. Their paper from 2018 is pretty comprehensive as they knocked out a many of the enzymes involved in the HS/sulfation pathway. There needs to be an explanation for why the double KO does not lead to a “cumulative” (as mentioned in the discussion).

As mentioned above in response to R#1, a total of 13 mutant cell lines were tested by the co-author (Lianchun Wang) for structural functional relationship of HS (PMID: 30377379). Several of these cell-lines are mutated for enzymes that are required for synthesis of basic HS structures. We hypothesized that specific sulfation of HS moieties is required for CMV entry. Thus we initially analyzed a subset of these mutants that perturb specific sulfation of HS and have been implicated earlier in HSV-1 entry (Fig 4). Later on we tested the entire library but only selected mutant cell lines survived the virus infection. These cell lines are represented in Fig 5. The comparisons have been made with the wild type cells in both cases and no claims have been made regarding the relative importance of one mutation over another. In fig 8 (now fig 4), all mutants were found to significantly impact virus titers compared to the wild-type and in fig 9 (now fig 5), all tested mutants were found to impact HCMV entry. It is important to note that there are at least six known isoforms of Hs3st and we have tested only 1, 4 and a double knock-out. Thus double KO may not necessarily have a cumulative effect as other isoforms may complement.

The switching and in complete analysis between HCMV and MCMV is confusing. Complete the analysis in either one or the other. Much of the previous works has been completed for HCMV so a complete analysis of MCMV entry would be more novel.

We initially worked on the manuscript from HCMV point of view since it’s clinically relevant; however, only mice mutant cell lines were available for studying, thus we switched to MCMV. We now included data on HCMV gB binding to heparin. Since gB is highly conserved among all CMVs we do not expect the results to be very different in MCMV.

An interesting concept that could make the manuscript more complete/interesting would be the inhibition experiments with endothelial, fibroblast, macrophage-tropic viruses and how they are or are not interfered with the dp HS/sulfations.

As mentioned above, different tropic strains of HCMV are known to differ in the presence or absence of a pentameric glycoprotein complex. However, glycoprotein B is highly conserved across HCMV strains. Our new data shows strong binding of heparin to gB confirming earlier studies with HSV-1. We designed the gB construct based on a clinical (Merlin) strain of HCMV.

Fig. 9 This is unconvincing and includes mutant lines not described/used in Fig. 8. There are potentially interesting cell line mutants that could provide interesting information if carried out as in Fig. 8’s set up. It would be better to use a GFP expressing virus to address entry instead of these western blots.

We mentioned why we used a selected set of these mutant cell lines above. IE1 expression in a western blot has been used as a surrogate for virus entry in numerous CMV studies. We used a GFP expressing virus to screen GAGs in Fig 2.

Fig. 10 The number of foci/well are too low. Usually (for consistency) you want closer to 50 /well. There are many different types of HS binding peptides. There were from Tiwari et al but there are others out there that block entry more generally and would be good to use as a positive/negative control. They would need to show the specificity of theses peptides on the cell types used. Why not see if they block entry on the different mouse KO lines? Would it further decrease entry/infection?

We have removed the peptide studies since they did not contribute significantly to the manuscript.

Should be using STD not SEM for figures. Also should show each data point to help in the interpretation.

We have re-analyzed several of the previous data-sets and plotted them again to address the issues with statistics.

Reviewer #2: (No Response)

Reviewer #3: • Line 70. Didn’t Hetzenecker 2015 show that HCMV can also use endocytosis pathways in fibroblasts?

Thanks for pointing this out. We have changed the sentence to read ‘Interestingly, human cytomegalovirus (HCMV) entry **mainly** follows direct fusion at the cell surface in fibroblasts, while entry into other relevant cell types, such as endothelial cells, follows an endocytic route’

• Fig2. Is there some kind of statistical error/significance test that can be used? The legend indicates six replicates, which implies that the values plotted are averages, is this the case? Fig 3 is the same type of analysis and has error bars, although the legend does not state what they represent. In any case, some analysis beyond standard deviation or standard error would likely be called for here.

Yes, the mean values are plotted. We have included error bars in both panels of new Fig. 1 and used standard deviation instead of ‘standard error of mean’ as error bars. Data was analyzed by ordinary one way ANOVA with multiple comparisons comparing the means of each test with control, and corrected using Dunnett’s post hoc test, showing significant differences among means (p <0.0001).

• Materials and methods indicates that HCMV Towne strain was used. Could the authors provide more information. Is this BAC derived? Or the ATCC Towne? (which is a mix of genotypes, right? Long and short at least?). Or is this some other variant of Towne? Moreover, given the explosion of research relating to HCMV genetic diversity, how can the authors conclude that this heparin interaction is characteristic of all strains or variants of HCMV? Particularly since there is no indication of what CMV glycoproteins are binding these GAGs (assuming this conclusion is true: see other comments).

The Towne strain we used was BAC-derived. This has been added to the materials and methods. Please see our response above related to gB-heparin binding.

• Line 249: I understand the point of this control, but if it is a negative control, then why name it a positive control only to have to explain here?

Corrected.

• Fig 4. It is unclear from the results text or the legend if the GAG derivatives were present throughout the 5 day experiment. Maybe it is in the materials and methods, but it is important enough to be stated in the legend. Is this testing initial infection (entry) or also subsequent spread over 5 days? Again, some statistical analysis beyond standard error would be good, especially since the data are normalized to %. What was the dynamic range? (how many foci were counted in the control wells?

We have added additional details about this assay in the legends as well as in material and methods. (Fig. 2 and Line 184)

• Fig 5 seems to be the same experiment as in Fig 4, but with an alternative readout (viral titer recovered at 5 days vs # of GFP+ foci at 5 days). What is the point of the plot in 5B? Is seems to be only a subset of the exact data from 5A, just plotted as dots connected by a line rather than as columns. If you are trying to show a correlation with the degree of polymerization, there are specific correlation tests that may be appropriate. The more interesting comparison would seem to be between the two readouts; ie, why does the titer recovered at 5 days not track with the GFP readout? If the GAG derivatives are present for the entire experiment, are the progeny release to the supernatant affected? Does this effect then carry over to the plaque assay titration? Or are the cells themselves affected by the GAGs?

Fig 4 (now fig 2) was a preliminary screening assay and differed from other assays not only in terms of assayed time points but the entity being assayed (GFP expression vs. total virus pfus recovered). We also measured cell cytotoxicity of GAGs (Fig. S4). The conclusions of the manuscript were based on several different types of experiments (glycoarrays, virus GFP inhibition assays, virus titers, mutant cell line virus entry, and protein binding assays.)

Also see this comment above:

“The results of the original Fig. 4 and Fig. 5 (now Fig. 2 and 3) actually indicate the opposite of what the reviewer infers. The major point we wanted to make with the current Fig. 3 is that virus inhibition was not due to a simple increase in the number of potential HCMV binding sites per molecule of GAG. This can be observed by comparing results from Fig. 3 A and B (where the amount of available negative charge varies directly with degree of polymerization) with those from Fig. 3 C and D (where the amount of available negative charge is constant, as all doses are at equivalent g/L rather than mol/L). As evidenced by similar trend of inhibition obtained when treating cells with equivalent µM or g/L concentrations of GAGs, the simple amount of negative charge available does not explain the decrease in titer at increased degrees in polymerization. A preferred explanation is a minimum ligand binding size needed to inhibit HCMV growth, and/or avidity effects from multiple binding sites increasing the apparent affinity of GAG chains able to engage more than one site. Additionally, our data indicate that 6O-sulfation is less important to HCMV binding than N-sulfation, even though the charge density is similar between to two selectively desulfated products. These points have been expanded in the discussion. We wanted to emphasize the trend rather than differences between individual treatments, thus the data has been analyzed by ordinary one way ANOVA with multiple comparisons comparing the means of each test sample with control, and corrected using Dunnett’s post hoc test. Differences were considered significant if p<0.05. Fig 4 (now Fig. 2) showed preliminary screening using virus-GFP as a marker, which is indicative of virus entry, whereas Fig. 5 (now Fig 3) analyzed virus titers to show an overall impact on virus replication.”

• Fig 7A. Vital dye exclusion is a very coarse assessment of potential effects of the GAGs on the cells. Is it possible that the physiology of the cell is altered in a way that does not “kill” the cell?

Potential effects of GAGs on the cell physiology continue to be characterized (See Wang et. Al. Nature Methods). We were most concerned with cell viability in uninfected and infected cell settings for the purpose of this manuscript.

• Line 294/Fig7B. Statement in results does not seem to match data shown.

We have changed the language to match the data in Fig. S4. “heparin (PIHSS) and some enoxaparin derivatives (*dp* 12 or greater) appeared to protect cells from death that was evident in mock-treated controls (Fig. S4B).”

• Fig 8. Legend states that the error bars represent standard error, but there is no indication of the statistical tool used for the “asterisks” of significance. If these asterisks are only indicating which error bars do not overlap with the control, they should be removed and an appropriate test used. (Probably one-way ANOVA+Dunnetts post hoc). Some of the differences seem quite small.

We have now included the description of statistical analysis in figure legends as well as in the materials and methods. Data was analyzed by ordinary one way ANOVA with multiple comparisons comparing the means of each test sample with control, and corrected using Dunnett’s post hoc test. Differences were considered significant if p<0.05.

• Fig 9. It would be much easier to interpret these data if the lane order in the blots matched the order of the relative density columns left to right.

We have rearranged the relative density columns to match the blot (Fig. 5).

• Lines 315-318. Is it really fair to say that the data in Fig 9 indicate differences in “entry?” I grant you that the notion suggested above that the GAG derivatives might affect cell physiology is a bit of the “formal caveat” (meaning it is formally possible, but maybe not all that likely). But here in fig 9, the cells have been asked to grow without the normal GAGs modification pathways. This, by definition, means their physiology is altered, no? I’m no expert on glycan modifications, but seems to me that the pathways are critical to the cell being able to move membrane proteins from the ER to the plasma membrane and then recycle them. It’s hard to imaging these cells are not physiologically abnormal. Maybe they just can’t support IE expression very well. Even if the cells are able to support IE expression normally after entry, how would you know that the entry defect is due to the lack of binding to the GAGs and not that the altered glycan processing pathways impacted the expression of specific receptors (if this was an HCMV experiment, I would suggest PDGFR-alpha, for example)?

Very insightful comment and a difficult task to prove if the cellular transport pathways were really altered in these mutant cells. Dr. Wang (co-author) has characterized these cells to a good extent. The data in fig 9 (now fig 5) is supportive of the data from other experiments in the manuscript and thus would not affect the overall conclusions even if an altered expression was the case.

• Line322. Is there some citation or supplemental data to support the specificity of these peptides as stated?

We have removed the peptide experiments as mentioned above.

• Fig 10. As suggested above, is it possible that these peptides alter cell physiology? In fact, this seems likely in this experiment since they are designed to actually bind to the cells, and may well engage signaling pathways similar to the natural ligands for the GAGs as indicated by the authors in Lines 42-43 (cytokines; growth factors etc).

We have removed the peptide experiments as mentioned above.
